# Human umbilical cord mesenchymal stem cells recover chemotherapy-induced premature ovarian failure

**DOI:** 10.3389/fmed.2025.1681233

**Published:** 2025-10-03

**Authors:** Guojie Ji, Pengbo Wang, Zhihong Kong, Xiangxiang Cao, Zhibin Sun, Jie Yang, Xiaoyang Zhao, Huigen Feng, Huanhuan Hu

**Affiliations:** ^1^Key Laboratory of Fertility Preservation, North Henan Medical University, Xinxiang, Henan Province, China; ^2^Zhengzhou Immuno Biotech Co., Ltd., Zhengzhou, Henan Province, China; ^3^School of Life Sciences and Technology, Xinxiang Medical University, Xinxiang, Henan Province, China

**Keywords:** premature ovarian failure, human umbilical cord mesenchymal stem cells, transcriptome sequencing, cyclophosphamide, busulfan

## Abstract

**Background:**

Current therapeutic approaches for premature ovarian failure (POF) are often inadequate in clinical practice and some raise ethical concerns. Human umbilical cord mesenchymal stem cells (hUC-MSCs) have emerged as a preferred option for cell transplantation, attributed to their facile extraction process and minimal immunogenicity.

**Objective and methods:**

This study aimed to elucidate the transcriptomic alterations associated with hUC-MSCs treated in the context of POF mice and to explore their underlying biological mechanisms. POF mice were established via injecting cyclophosphamide (CTX) plus busulfan (BU). Subsequently, ovaries and serum were collected after 1 week for model identification. 4 weeks after hUC-MSCs transplantation, ovaries and serum were collected for experimental analysis. Differentially expressed genes (DEGs) were identified using RNA sequencing (RNA-seq), and their expression levels were validated through reverse transcription quantitative polymerase chain reaction (RT-qPCR).

**Results:**

After hUC-MSCs therapy, the number of follicles recovered significantly and the atretic follicles decreased significantly. FSH was reduced, AMH and E2 levels were increased in the treatment group, and comparable to the control group. 343 DEGs were detected in the POF group and the treatment group, including 187 up-regulated genes and 156 down-regulated genes. Our comparative analysis of hUC-MSCs treated with POF samples revealed significant involvement of biological pathways and processes related to cell adhesion, proliferation, apoptosis, inflammatory response and immune response.

**Conclusion:**

Our research offers a novel perspective on the application of hUC-MSCs for the treatment of POF and establishes a foundation for further exploration of their potential clinical applications.

## 1 Introduction

Premature ovarian failure (POF), refers to a disease characterized by non-physiological amenorrhea, elevated follicle-stimulating hormone levels, and estrogen deficiency in women after puberty and before age 40. It is one of the leading causes of female infertility (1). The reported incidence of POF in women is 0.01% by age 20, 0.1% by age 30, and 1% by age 40. Recently, the incidence of POF has been increasing (2). POF is a heterogeneous disease with diverse pathogenesis, mainly caused by chromosomal aberrations, genetic diseases, autoimmune disorders, metabolic abnormalities, environment and chemoradiotherapy (3).

The current conventional treatment approaches for POF include hormonal replacement therapy, melatonin supplementation, immunomodulatory therapy, and cells transplantation (3). However, existing POF treatments are less effective and may lead to significant side effects such as increased cardiovascular disease, osteoporosis, sexual dysfunction, stroke, venous thrombosis, endometrial, breast cancers, ovarian cancers and the risk of other related diseases (4).

Mesenchymal stem cells (MSCs) are pluripotent stem cells capable of self-renewal and multi-directional differentiation. MSCs come from a wide range of sources, such as umbilical cord, bone marrow, fat, ovary, peritoneum, amniotic membrane, menstrual blood and placenta (5–8). MSCs are widely used in regenerative medicine owing to their abundant sources and no ethical controversy (9). Studies have demonstrated that umbilical cord mesenchymal stem cells (UC-MSCs) can differentiate into oocyte-like structures and endometrial cells and express germ cell-specific mRNA and protein markers (10). In 2018, the world’s first clinical research on the treatment of POF with MSCs composite scaffolds achieved significant results, with two patients achieving clinical pregnancy and the birth of the first healthy baby (11). Intraovarian injection of menstrual blood-derived mesenchymal stem cells (MB-MSCs) improved the endocrine of POF patients, and a few patients recovered menstruation (12). In animal POF models and human POF patients, studies have verified that UC-MSCs can promote stem cell renewal, activate primordial follicles, enhance ovarian function and reduce ovarian cell death through paracrine action, thus repair ovarian function to restore reproductive function and fertility (13, 14). Further studies have demonstrated that UC-MSCs may affect mitogen-activated protein kinase (MAPK) signaling pathway, G protein-coupled receptor (GPCR) signaling pathway, insulin signaling pathway and regulation of key factors that induce apoptosis reduce the apoptosis of Granulosa cells (GCs) (2, 15).

Other studies have found that UC-MSCs on collagen scaffolds activate primordial follicles by phosphorylating transcription factors Forkhead Box protein O1 (FoxO1) and FoxO3a (16). In addition, UC-MSCs were observed to induce the production of angiogenic growth factors such as Vascular Endothelial Growth Factor VEGF, HGF, PGF and TGF-β1, and increase cell proliferation and vascular marker expression to improve endometrial damage and infertility (2, 16, 17).

In this study, a mouse model of POF was induced by chemotherapeutic drugs, and hUC-MSCs were transplanted for treatment. Transcriptome analysis of mouse ovaries in the POF group and the treatment group was performed to screen genes associated with the hUC-MSCs treated of POF in order to provide support for related studies.

## 2 Materials and methods

### 2.1 Isolation of hUC-MSCs

This study was approved by the Ethics Committee of North Henan Medical University, after obtaining the consent of the mother and her family through the affiliated hospital, sterile collection of neonatal umbilical cord (≥15 cm) during full-term cesarean section. Wharton’s jelly was removed aseptically and cut into tissue blocks of about 1 mm^3^, which were uniformly inoculated into T75 culture bottle and cultured overnight with 5 mL complete culture medium (2% Ultra-CULTURE+ 5% L-glutamine). After the tissue block is firmly attached to the wall, replace the complete medium and change the medium every 3 days. After sufficient hUC-MSCs grew around the tissue mass, pancreatic enzyme digestion and passage were performed, the second generation of hUC-MSCs were harvested for the experiment.

### 2.2 Flow cytometry analysis

According to previous study (10, 18), the surface markers were detected by flow cytometry. The expression of cell surface markers CD73, CD90, CD105, CD34, CD45, CD11b, CD19 and HLA-DR in hUC-MSCs were analyzed by flow cytometry. Collected hUC-MSCs were incubated with antibodies at 4 °C for 30 min. Subsequently, the cells were washed with 0.9% sodium chloride solution, centrifuged and resuspended. Flow cytometry assay was performed by fluorescence-activated cell sorting (FACS) and the results were analyzed with FlowJo software.

### 2.3 Experimental animals and living conditions

Specific pathogen-free female KM mice (6–8 weeks) were obtained from Vital River Laboratory Animal Technology Co., Ltd. (Beijing, China). Mice were fed in the specific pathogen-free condition with constant temperature and humidity on a 12–12 h light–dark cycle and free access to food and water. All animal experiments were approved by the Ethical Committee of North Henan Medical University.

### 2.4 POF mouse model

Thirty female mice were randomly divided into two groups: control group (*n* = 10) and the POF group (*n* = 20). Next, The mice in the POF group were intraperitoneally injected with a single dose of CTX (120 mg/kg) and BU (30 mg/kg) (*n* = 10) (19). To confirm successful establishment of POF mice, mouse activity, diet, body weight, hair changes, estrous cyclicity, ovarian weight, serum hormone and morphological characteristics of ovary were examined at 1 week after modeling.

### 2.5 hUC-MSCs transplantation

The second-generation hUC-MSCs are preserved in liquid nitrogen in the laboratory. hUC-MSCs were stored in 0.9% saline after activation. The treatment group was injected with 200 μL 3 × 10^6^ cells/mL of hUC-MSCs via the tail vein after modeling for 1 week. For comparison, the same volume of 0.9% sodium chloride solution was injected into POF group and control group mice.

### 2.6 Quantitative real-time polymerase chain reaction (RT-qPCR) analysis

Total RNA was isolated and purified using TRIzol reagent (Ambion, USA) in accordance with the manufacturer’s instructions (20). RNA of each sample was quantified using Nanodrop ND-1000 (Nanodrop, Wilmington, DE, USA). RNA integrity was confirmed by electrophoresis with a denaturing agarose gel. RT-qPCR analysis were performed as described previously (21). The primers used for quantification were designed using PrimerBLAST on the NCBI website.^1^ Each sample was repeated thrice, and relative expression levels were calculated using the 2^−ΔΔCt^ method, and *GAPDH* was used for control. Primer sequences were provided in Supplementary Table S1.

### 2.7 mRNA library construction and transcriptomic analysis

According to the intra-group correlation and inter-group difference of mRNA expression profiles in each group, the transcriptional data of the POF group and treatment group were used for bioinformatics analysis (22). RNA libraries were sequenced on the Illumina sequencing platform by LC-BIO Co., Ltd. Cutadapt software (version: 1.9) was used to remove the reads that contained adaptor contamination. And after removed the low quality bases and undetermined bases, we used HISAT2 software (version: 2-2.0.4) to map reads to the genome (Mus Ensembl v101). The mapped reads of each sample were assembled using StringTie (version: 1.3.4d) with default parameters. Then, all transcriptomes from all samples were merged to reconstruct a comprehensive transcriptome using gffcompare software (version: 0.9.8). After the final transcriptome was generated, StringTie and ballgown were used to estimate the expression levels of all transcripts and perform expression level for mRNAs by calculating FPKM. The differentially expressed mRNAs were selected with fold change >2 or fold change <0.5 and *p* < 0.05 by R package DESeq2, and then analysis GO enrichment and KEGG enrichment to the differentially expressed mRNAs.

### 2.8 Statistical analysis

Statistical analyses were performed using GraphPad Prism 9.0. All results were obtained from at least three biological replicates and were presented as means ± SEM. Differences between means were analyzed using *t*-test. *p* < 0.05 was regarded as statistically significant. For the *in vitro* experiments, each group set had three samples and each sample set had three replications. For the *in vivo* experiments, each group set contained ten mice.

## 3 Results

### 3.1 Isolation, culture and identification of hUC-MSCs

**Figure 1:**
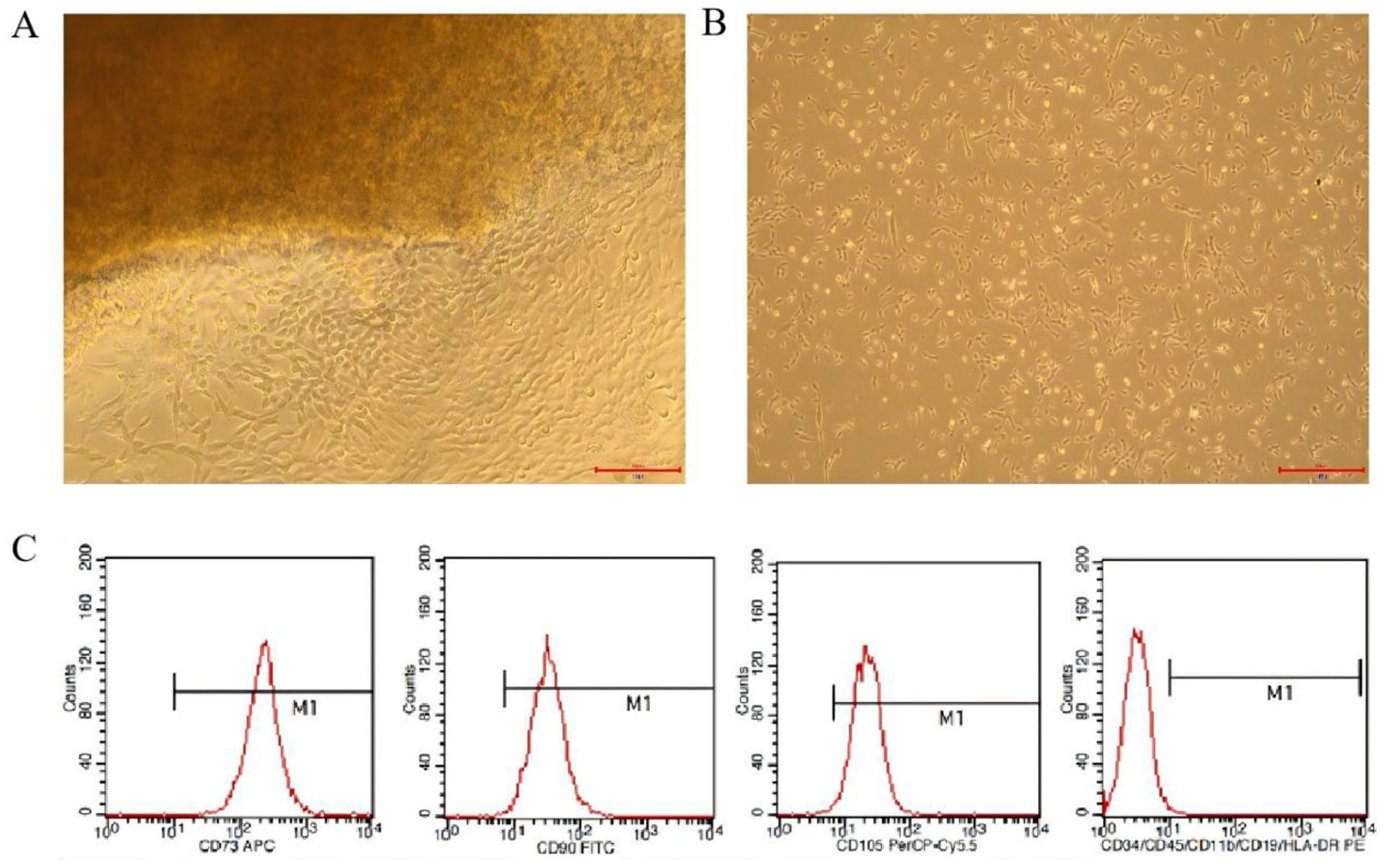
Isolation, culture and identification of hUC-MSCs. **(A)** Primary hUC-MSCs morphology. **(B)** Passage 2 hUC-MSCs morphology. **(C)** Flow cytometry analysis results of hUC-MSCs.

A large number of cells crawled out 5 days after the tissue block was attached to the wall (Figure 1A). After passage, the cells were mainly spindle-shaped, translucent, with clear outline and good refraction (Figure 1B). hUC-MSCs stable height table up to CD73, CD90, CD105; CD34, CD45, CD11b, CD19 and HLA-DR were rarely expressed (Figure 1C). This indicated that the hUC-MSCs obtained from the experiment were in accordance with the International Cell Therapy Association (ISCT) mesenchymal stem cell standard.

### 3.2 CTX combined with BU leads to ovarian dysfunction

**Figure 2:**
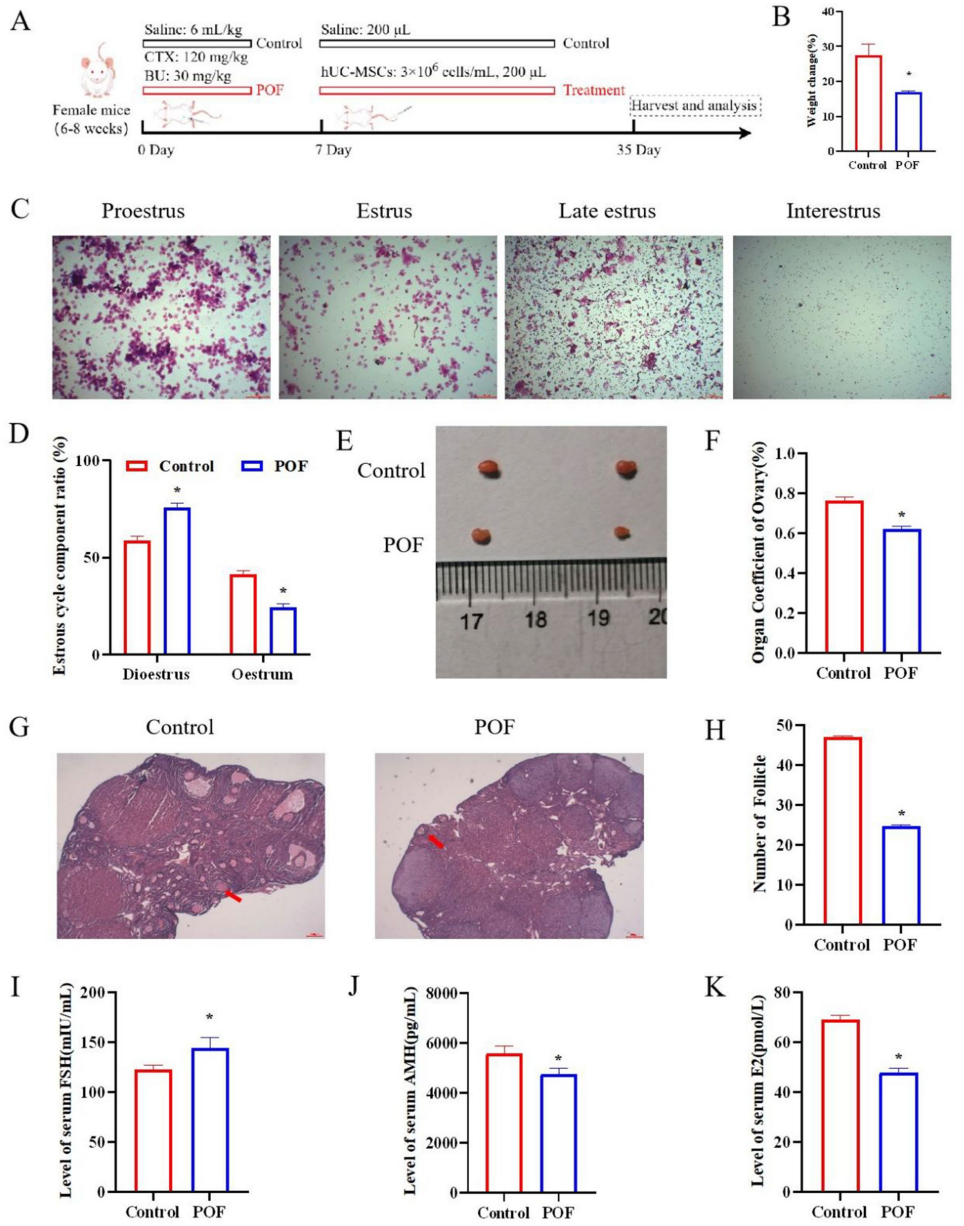
Effect of CTX combined with BU on ovary structure and function in mice. **(A)** Animal experiment design. Mice were intraperitoneally injected with CTX (120 mg/kg) and BU (30 mg/kg), 1 week later, hUC-MSCs (3×10^6^ cells/mL, 200 μL) were injected via the tail vein, while control mice were given saline. **(B)** Changes in the body weight of mice in each group during the experiment. **(C,F)** The estrous cycle of mice. Proestrus: the nucleated cells are round, the nucleus is dyed purple red, the cytoplasm is dyed rose red, and there are rose red non-nucleated keratinocytes; estrus: rose red keratinized cells, irregular polygon shape; Late estrus: a large number of white blood cells and a small number of non-nuclear keratinocytes; Interestrus: leukocytes, nucleated cells and non-nucleated keratinocytes exist simultaneously. **(D,H)** H&E staining and number of follicles. **(E,G)** Representative images of ovary and organ coefficient. **(I–K)** Serum E2, FSH and AMH content in control and POF mice. Data are presented as mean ± SEM (*n* = 3). *p < 0.05 vs. the control group.

The POF mouse model was established to study the toxic effects of CTX combined with BU on ovaries. The KM mice were treated with CTX and BU (Figure 2A). After 1 week, the mice showed weight loss (Figure 2B), which is an important indicator of the health of the mice. Result shows representative images of each period of the estrous cycle (Figure 2C), the estrus interval in POF group was significantly longer compared with the control group (Figure 2D). At the same time, ovarian atrophy and contraction were found (Figure 2E), and the ovarian organ coefficient in the POF group was significantly lower than that in the control group (Figure 2F). H&E staining showed that the number of atretic follicles increased and the number of follicles decreased in POF group (Figures 2G,H). Elisa results showed that FSH levels was significantly increased, AMH and E2 levels were significantly reduced in the POF group (Figures 2I–K).

### 3.3 hUC-MSCs alleviates ovary injury

**Figure 3:**
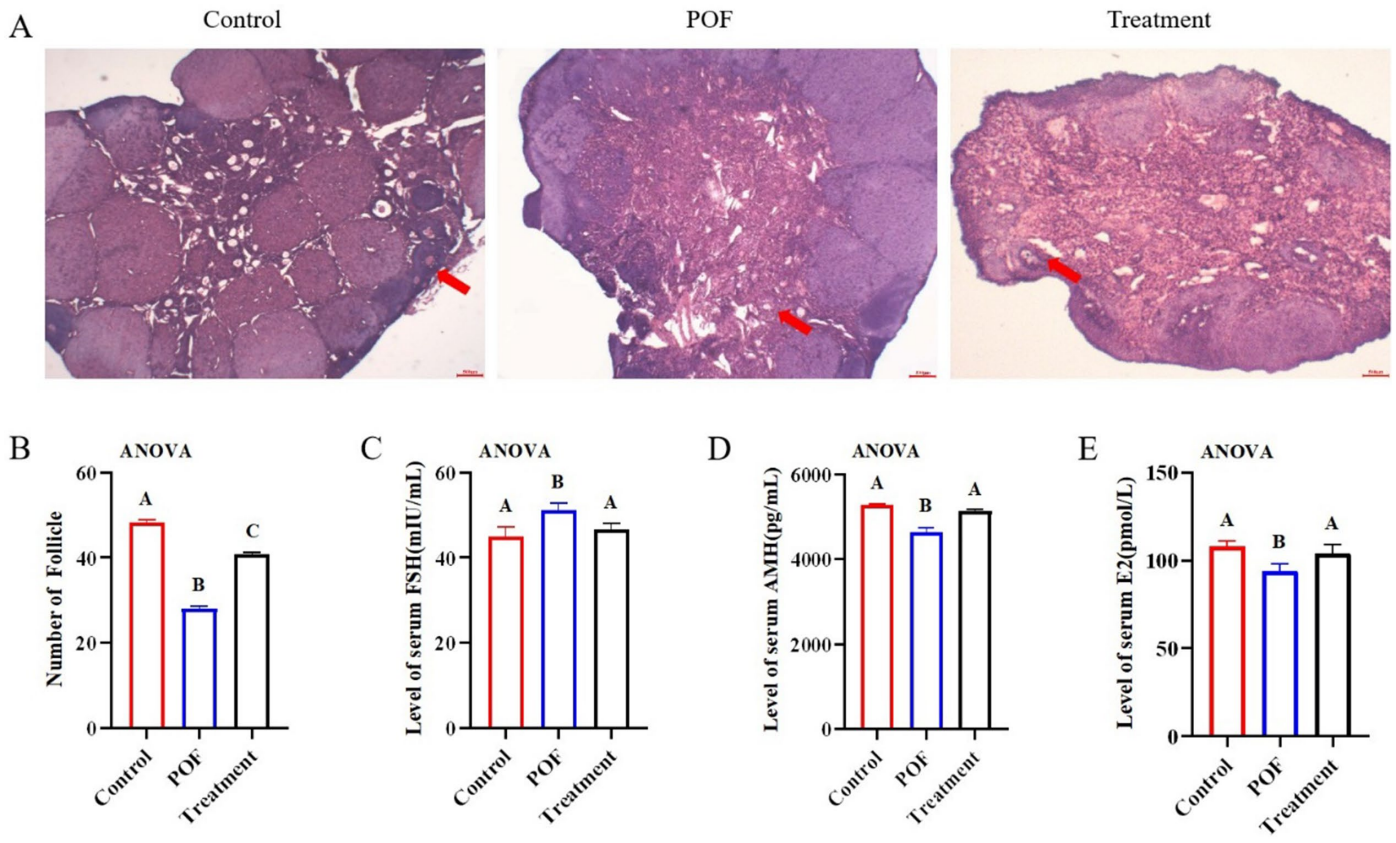
hUC-MSCs alleviates ovary injury. **(A,B)** Ovarian H&E staining and number of follicles in each group. **(C–E)** Serum E2, FSH and AMH content in control and POF mice. Data are presented using ANOVA (*n* = 3). *p < 0.05, ***p < 0.001, ****p < 0.0001.

To verify the effect of hUC-MSCs on ovarian function in POF mice, ovarian tissue recovery and serum follicle stimulating hormone (FSH), anti-Mullerian hormone (AMH) and estrogen (E2) levels were measured by H&E staining and Elisa. H&E staining showed that the number of follicles recovered significantly and the atretic follicles decreased significantly (Figures 3A,B). Elisa results showed that FSH was significantly reduced, AMH and E2 levels were significantly increased in the treatment group, and there was no significant difference compared with the control group (Figures 3C–E).

### 3.4 Differential gene expression

**Figure 4:**
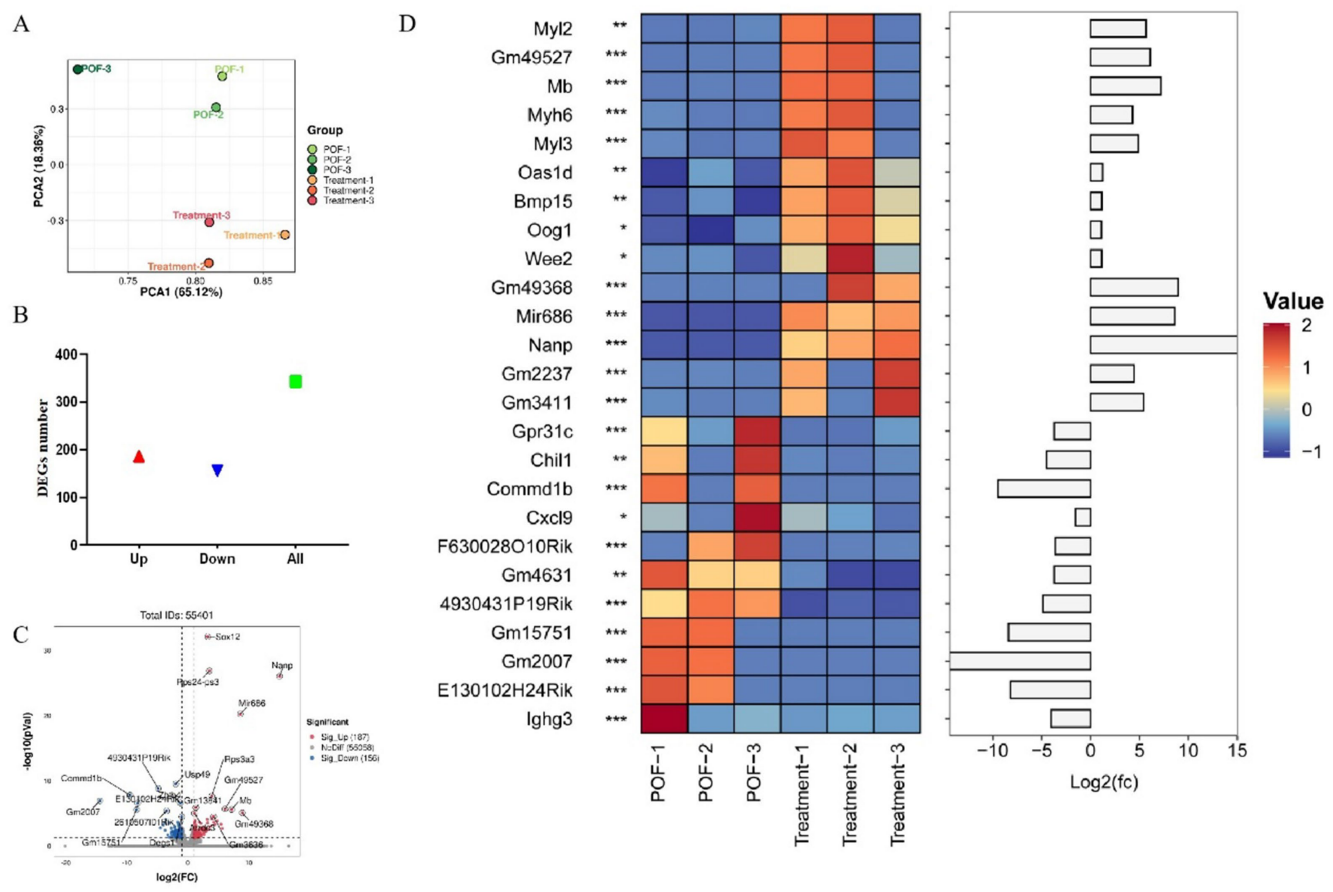
DEGs analysis based on RNA sequencing of mouse ovary. **(A)** PCA analysis of RNA sequencing in each group. **(B)** Histogram of up or down-regulated DEGs. **(C)** Volcano map of DEGs screening based on RNA sequencing. **(D)** Heat map of relative mRNA levels of relative genes of mouse ovary.

To identify genes involved in the recovery of ovarian function, genome-wide expression analysis of ovarian tissues was performed in the POF group and treatment group. DEGs are screened with |logFC| > 1 and *p* < 0.05. Results of the principal component analysis (PCA) is showed good intergroup consistency (Figure 4A). A total of 343 DEGs were identified, including 187 up-regulated genes and 156 down-regulated genes (Figure 4B; Supplementary Table S2), Up-regulated genes include Nanp, Gm49368, Mir686, Mb, Gm49527, Myl2, Gm3411, Myl3, Gm2237, Myh6, Oas1d, Bmp15, Wee2, and Oog1, as well as down-regulated genes include Gm2007, Commd1b, Gm15751, E130102H24Rik, 4930431P19Rik, Chil1, Ighg3, Gm4631, Gpr31c, F630028O10Rik, and Cxcl9. DEGs were identified by threshold were visualized by volcano plot, red and blue dots indicated up-regulated and down-regulated genes, respectively, with all DEGs’ expression levels shown in the heatmap (Figures 4C,D).

### 3.5 GO enrichment analysis of DEGs

**Figure 5:**
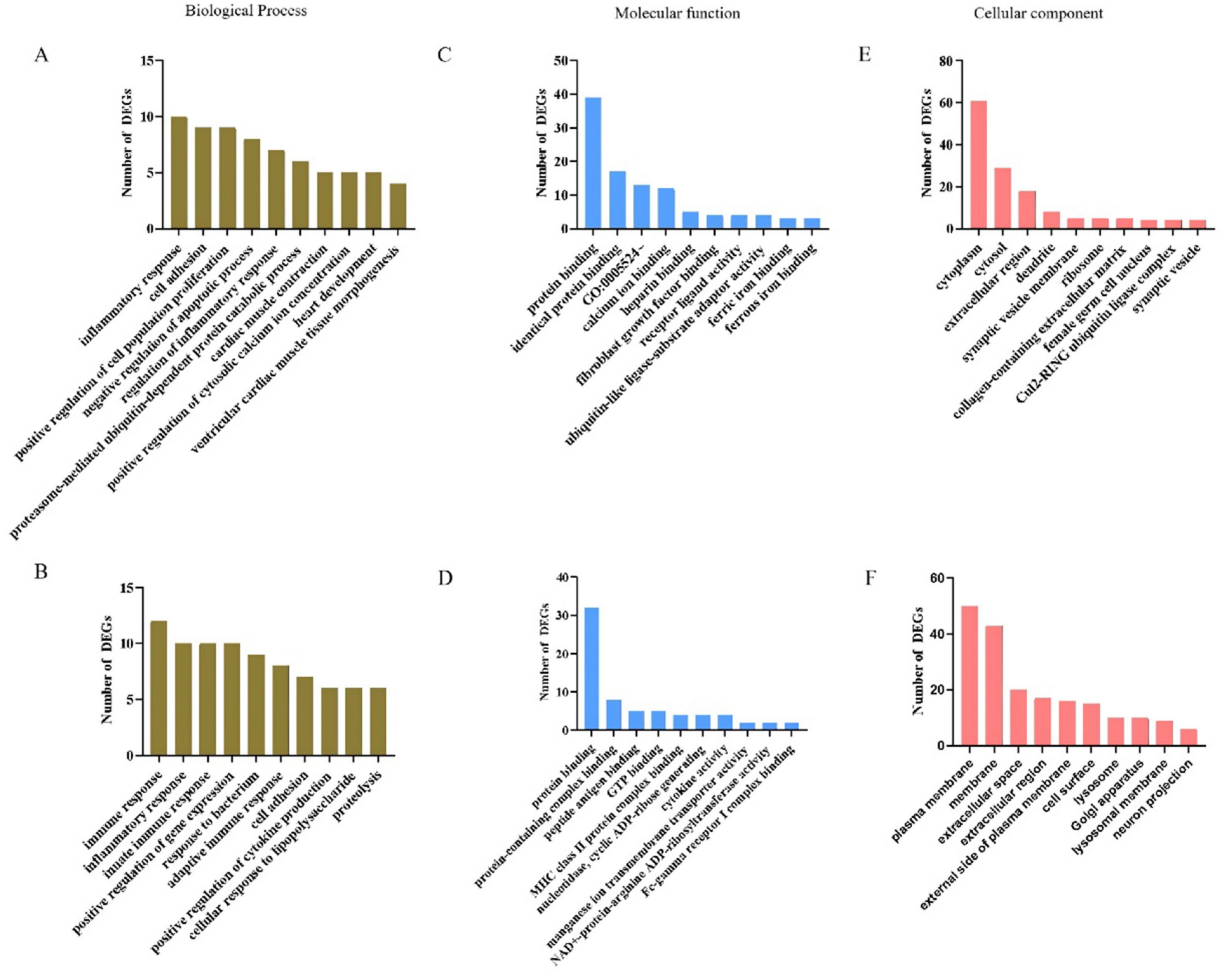
GO enrichment analysis for biological process, molecular function and cellular component of DEGs. **(A–C)** Top10 GO terms of up-regulated differential genes. **(D–F)** Top10 of GO terms of down-regulated differential genes.

The GO framework comprises three categories: biological process (BP), molecular function (MF) and cellular component (CC). GO BP analysis indicated that up-regulated genes were significantly enriched in processes such as inflammatory response, cell adhesion, positive regulation of cell population proliferation and negative regulation of apoptotic processes (Figure 5A; Supplementary Table S3). Conversely, down-regulated genes were significantly enriched in immune response, inflammatory response, innate immune response, adaptive immune response and cell adhesion (Figure 5B; Supplementary Table S4). In the analysis of MF terms, the up-regulated genes were significantly enriched in categories such as protein binding, identical protein binding, ATP binding, fibroblast growth factor binding, receptor ligand activity and ubiquitin-like ligase-substrate adaptor activity (Figure 5C; Supplementary Table S3). Conversely, the down-regulated genes showed significant enrichment in MF terms including protein binding, protein-containing complex binding, peptide antigen binding, MHC class II protein complex binding and Fc-gamma receptor I complex binding (Figure 5D; Supplementary Table S4). Regarding the GO CC analysis, the up-regulated genes were enriched in terms such as cytoplasm, cytosol, extracellular region, collagen-containing extracellular matrix, female germ cell nucleus and Cul2-RING ubiquitin ligase complex (Figure 5E; Supplementary Table S3). In contrast, the down-regulated genes were enriched in terms associated with the plasma membrane, membrane, external side of plasma membrane, cell surface, lysosome, Golgi apparatus and lysosomal membrane (Figure 5F; Supplementary Table S4).

### 3.6 DEGs signaling pathway and protein interaction analysis

**Figure 6:**
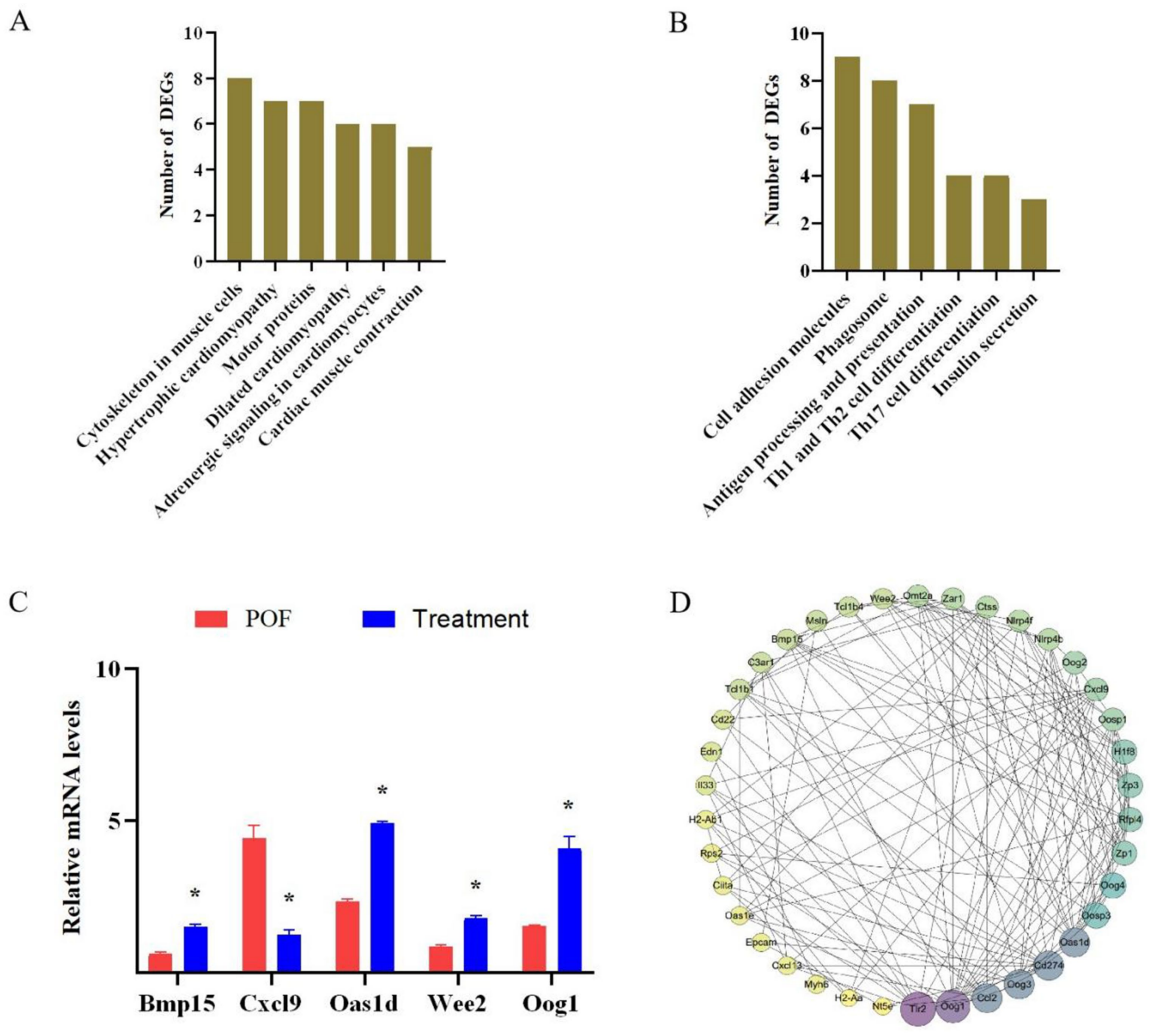
DEGs signaling pathway and protein interaction analysis. **(A)** KEGG enrichment analysis of up-regulated genes. **(B)** KEGG enrichment analysis of down-regulated genes. **(C)** RT-qPCR verified RNA sequencing. **(D)** Proteins-proteins interaction analysis. *p < 0.05 vs. the POF group.

To elucidate the distinct pathways of DEGs in the POF and treatment groups, a KEGG enrichment analysis was conducted. The analysis revealed that up-regulated genes were significantly enriched in pathways related to cytoskeleton in muscle cells, motor proteins, and Cardiac muscle contraction (Figure 6A). Conversely, down-regulated genes were predominantly associated with pathways involving the Cell adhesion molecules, phagosome, antigen processing and presentation, Th1 and Th2 cell differentiation, Th17 cell differentiation, and Insulin secretion (Figure 6B). These findings suggest a potential association between these signaling pathways and hUC-MSCs treatment. To validate the reliability of the transcriptome sequencing results, RT-qPCR was performed on five randomly selected genes. RT-qPCR results indicated significant up-regulation of Bmp15, Oas1d, Wee2 and Oog1, while Cxc19 was significantly down-regulated (Figure 6C). Thereby corroborating the accuracy of our transcriptome sequencing data. Furthermore, to explore the interactions among the 343 differentially expressed proteins, proteins-proteins interaction (PPI) network was constructed (Figure 6D; Supplementary Figure S1). A complex PPI network was identified, characterized by both direct and indirect interactions among the majority of proteins, as indicated by the number of observed nodes. Consistent with our previous findings, the following biological processes were significantly overrepresented: the immune system (17 proteins) and the regulation of cell proliferation and differentiation (6 proteins). Within the PPI network, certain proteins exhibited a high degree of connectivity. For instance, Oog1 demonstrated 18 interactions and was associated with proteins involved in the regulation of cell proliferation, apoptosis, differentiation, DNA-templated transcription and ubiquitin-dependent protein catabolic processes. Similarly, BMP15 exhibited 10 interactions and was linked to proteins involved in the regulation of DNA-templated transcription, the immune system and the regulation of tumor necrosis factor production.

## 4 Discussion

The clinical efficacy of the existing treatment methods for POF is not very satisfactory, and some methods still have ethical disputes. hUC-MSCs are easy to extract, have low immunogenicity, and have now become the first choice for cells transplantation. Transplantation of hUC-MSCs can reduce the number of atretic follicles and restore the ovulation cycle (19). It can also reduce the apoptosis of GCs by regulating autophagy, thereby improving ovarian function (21). However, a lack of knowledge of the molecular mechanism of hUC-MSCs in the treatment of POF. The purpose of this study was to identify transcriptomic alterations in hUC-MSCs treated POF. Transcriptome analysis was used to assess the relevant biological alterations, focusing on alterations in biological pathways and PPI networks that were not available in the current literature.

Our comparative analysis of hUC-MSCs treated with POF samples revealed significant involvement of biological pathways and processes related to cell adhesion, proliferation, apoptosis, inflammatory response and immune response. Notably, certain proteins, such as Oogenesin 1 (Oog1), were consistently upregulated and associated with cell proliferation, apoptosis and differentiation. The Oog protein family plays a crucial role in reproductive health and fertility by regulating various intracellular biological processes, particularly in the development of germ cells, including oocyte formation and maturation. The Oog family primarily comprises Oog-1, −2, −3 and −4 (23). Our study identified 4 previously described Oog genes, and enrichment analysis indicated their involvement in regulating cell proliferation and differentiation, potentially influencing chromosomal recombination during germ cell meiosis. Additionally, these genes participate in proteasome-mediated ubiquitin-dependent protein degradation, which may contribute to intracellular protein degradation and recycling, thereby impacting apoptosis. Oog1 gene is expressed in oocytes and early embryos, initiating expression on embryonic day 15.5 and localizing within the nucleus of oocytes (24, 25), this gene plays a crucial role in promoting the normal differentiation of female germ cells into oocytes by inhibiting the expression of genes associated with spermatogenesis (26).

PPI analyses have demonstrated that Oog1 directly interacts with proteins primarily involved in oocyte maturation and development, such as Bmp15, Oas1d, Oosp1, Oog2, Oog3, Oog4, Zar1, Rfpl4, Hif8, Omt2a, Zp1 and Zp3, as well as those involved in immune response processes, including Oas1d, Oas1e, Cd274, Ccl2, Nlrp4f and Nlrp4b. Proteins that directly interact with Bmp15, such as Oas1d, Wee2, Oog1, Zar1, Rfpl4, Hif8, Zp1 and Zp3, are predominantly engaged in the biological processes of oocyte maturation and development.

Bmp15 is a growth factor expressed in oocytes that plays a key role in ovarian function, primarily by regulating the growth of granulosa cells and the development of follicles, affecting the overall function of the ovary. Studies have shown that the loss of Bmp15 can lead to its dysfunction, which can trigger premature ovarian failure (27).

The expression of Oas1d may be related to the expression of Bmp15 and Oog1. Loss of Oas1d leads to impaired ovarian function, which in turn affects the expression and function of Bmp15 and Oog1, thereby affecting oocyte development and fertility (28). Oas1d is an oocyte-specific 2-5-oligoadenylate synthetase-like protein exhibiting 59% sequence homology with Oas1a. Expression of Oas1d is restricted to the ovaries, specifically localized within oocytes and the cytoplasm of early embryos, and is not detectable in later embryonic stages or other cell types. Knockout of the Oas1d gene in female mice results in decreased fertility, characterized by a reduced number of ovulations, impaired early embryonic development and increased embryonic fragmentation. Histological analyses indicate that Oas1d and Oas1a can interact both *in vivo* and *in vitro*, with Oas1d capable of inhibiting Oas1a enzymatic activity in a dose-dependent manner. It is hypothesized that Oas1d may inhibit the Oas1a-mediated interferon (IFN)/OAS/RNase L RNA degradation pathway. Functioning as a protective mechanism, this process prevents oocyte loss during viral infection, thereby preserving female fertility. The protective role of Oas1d in safeguarding oocytes and early embryos is crucial, as its absence leads to reproductive defects in mice (29–31).

Immune processes are implicated in the pathogenesis of numerous gynecological disorders, including POF, polycystic ovary syndrome (PCOS), endometriosis, and cervicitis. Previous studies have demonstrated immune dysregulation in both peripheral blood and the local ovarian microenvironment of POF patients, characterized by heightened Th1 cells responses, deficiencies in Tregs, and a significant correlation between the Th1/Treg ratio and POF severity. Interferon-gamma (IFN-*γ*) and tumor necrosis factor-alpha (TNF-*α*), secreted by Th1 cells, may impair ovarian function by activating immune responses, promoting inflammation, and inducing granulosa cell apoptosis (32). Research indicates that Th17 cells and their associated cytokines are markedly elevated in POF patients, suggesting their involvement in disease pathogenesis through pro-inflammatory effects and immune dysregulation, thereby affecting ovarian function (33–36). Furthermore, the PI3K/AKT/mTOR signaling pathway participates in the development of various autoimmune diseases by regulating Th17 cell differentiation. In POF, whether this signaling pathway exhibits abnormalities and its relationship with ovarian function remain unclear, warranting further investigation to provide novel insights into disease mechanisms (37).

Antigen processing and presentation are one of the core mechanisms by which the immune system recognizes and responds to foreign substances. This process involves degrading antigens (such as viral or bacterial proteins) into small molecular fragments, which are then bound to major histocompatibility complex (MHC) molecules to form antigenic peptide–MHC complexes. These complexes are subsequently presented to T cells, thereby activating adaptive immune responses (38). Phagosomes, while serving as critical components of innate immunity, bridge innate and adaptive immunities through antigen presentation. Their maturation and activation of signaling pathways promote T cell activation and initiation of adaptive immune responses (39). Cell adhesion not only participates in immune system functionality and regulation but also engages in diverse physiological and pathological processes—including immune cell recognition, migration, homing, and inflammatory responses. These processes rely on coordinated interactions among multiple adhesion molecules and signaling pathways, collectively maintaining immune homeostasis and normal function (40).

Motor proteins use energy generated from ATP hydrolysis, with their tail domains binding to the cytoskeleton to drive intracellular processes such as vesicle transport, cellular motility, and cell division. The cytoskeleton serves as both a sensor and mediator of apoptosis. Studies demonstrate that dynamic alterations in the cytoskeleton—including reorganization of actin filaments and microtubules—can modulate apoptotic signaling cascades and regulate the progression of apoptosis. Furthermore, the cytoskeleton influences both differentiation and functionality of follicular granulosa cells, while actively participating in oocyte maturation, chromosome segregation, organelle trafficking, and establishment of cellular polarity (41, 42).

In this study, only 10 mice were included in each group, which is consistent with the common design of preliminary exploratory studies, but there are limitations in clinical transformation. Although this study did not directly assess immune cell populations or associated cytokines. But we found a key role of immune regulation in POF treatment through transcriptome data analysis. However, whether the observed improvement in ovarian function-related indicators following treatment is associated with the regulation of immune dysregulation warrants further investigation. Subsequent studies can further improve the reliability and universality of the results by expanding the sample size, introducing independent validation cohorts (cell models). In order to further improve the reliability and generality of the results, qPCR was used to analyze the function of the key differential genes. MSCs require ISCT screening, patient stratification, and gene modification prevention and control due to inherent genetic abnormalities arising from prolonged culture or transplantation, as well as tumor-promoting effects. Different administration routes (intravenous and local delivery), are appropriate for varying symptoms. Low doses demonstrating limited efficacy and high doses potentially increasing adverse reactions. Therefore, these should be combined with pharmacodynamic markers and standardized detection. A longer follow-up period should focus on monitoring delayed adverse events, and cell trans differentiation, in accordance with EMA and FDA guidelines. In brief, MSCs therapy for POF necessitates the integration of mechanistic research and clinical application. The development of safe and effective MSC therapies should be expedited through head-to-head trials, companion diagnostics, and standardized production processes.

In conclusion, hUC-MSCs therapy restores endocrine, ovarian structure and function in POF mice, but remains a major challenge for clinical translation. Future research should prioritize: (1) Enlarging sample sizes to validate findings; (2) Refining treatment protocols to enhance therapeutic outcomes; and (3) Elucidating molecular mechanisms underlying treatment effects and their interplay with the immune system, to develop more effective clinical strategies for POF management. Concurrently, rigorous evaluation of long-term safety profiles and potential adverse effects remains imperative to maximize benefits for patients.

## Data Availability

The original contributions presented in the study are publicly available. This data can be found here: [https://www.ncbi.nlm.nih.gov/sra, PRJNA1331783].

## Footnotes

^1^ https://www.ncbi.nlm.nih.gov/tools/primer-blast/
