# Supplementary material for: Human umbilical cord mesenchymal stem cells recover chemotherapy-induced premature ovarian failure
Source: Front Med (Lausanne). 2025 Oct 3;12:1681233. doi: 10.3389/fmed.2025.1681233 (PMC12531164; doi:10.3389/fmed.2025.1681233)
Supplement: Supplementary file 1 [file Table_1.docx]

**Supplemental Materials**

**Table S1. Primer sequences**

| Gene name | Oligonucleotide sequence (5'-3') |
| --- | --- |
| *GAPDH-*F | AGGTCGGTGTGAACGGATTTG |
| *GAPDH-*R | TGTAGACCATGTAGTTGAGGTCA |
| *Usp49-*F | AGTTCCGGGAATGTTTCCTGA |
| *Usp49-*R | CTCCTTACTGACAACTCTGCG |
| *BMP15-*F | TCCTTGCTGACGACCCCTACAT |
| *BMP15-*R | TACCTCAGGGGATAGCCTTGG |
| *GPR75-*F | CTCAGGCTTCGTCATCATGTC |
| *GPR75-*R | AGGGTAAGGAGCAAGATGCAG |
| *NFAT5-*F | CAGCGCCCAATAGTTGGCA |
| *NFAT5-*R | TGCTGGTGAAAAATTGACTGGT |
| *OOG3-*F | CTTCTACAGCACACATCCAAGC |
| *OOG3-*R | TCCAGGAGTTCGGGACAAAAT |
