# Supplementary figures and images for: Human umbilical cord mesenchymal stem cells recover chemotherapy-induced premature ovarian failure
Source: Front Med (Lausanne). 2025 Oct 3;12:1681233. doi: 10.3389/fmed.2025.1681233 (PMC12531164; doi:10.3389/fmed.2025.1681233)

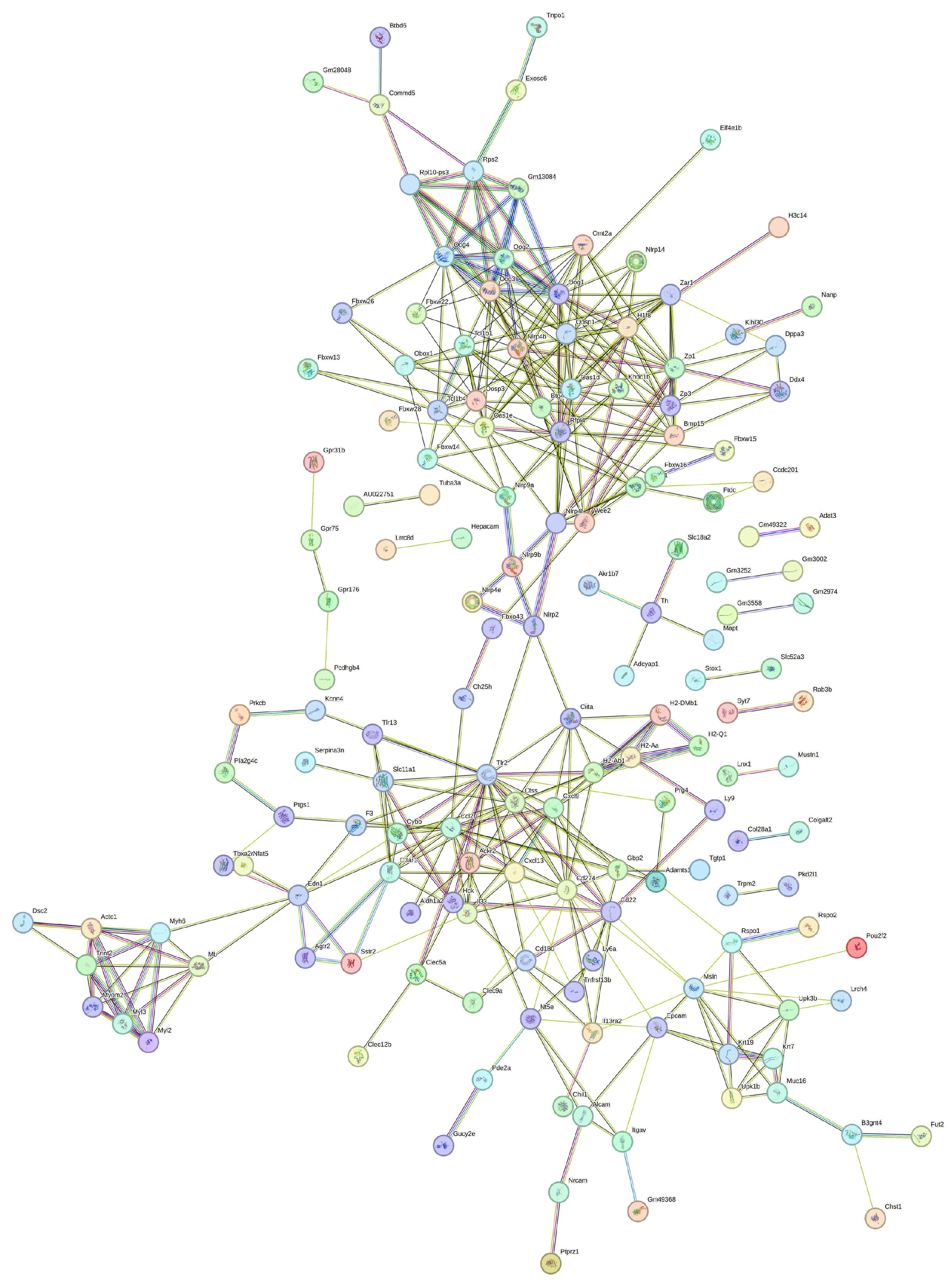

Supplement: Supplementary file 5 [file Image_1.jpeg]
